# Preparation and characterization of silver orthophosphate photocatalytic coating on glass substrate

**DOI:** 10.1038/s41598-021-93352-z

**Published:** 2021-07-07

**Authors:** Masahide Hagiri, Kenichi Uchida, Mika Kamo Sasaki, Shofiyah Sakinah

**Affiliations:** Department of Applied Chemistry and Biochemistry, Fukushima College, National Institute of Technology, Nagao 30, Kamiarakawa, Taira, Iwaki, Fukushima 970-8034 Japan

**Keywords:** Environmental sciences, Chemistry, Materials science, Physics

## Abstract

The photocatalytic activity of silver orthophosphate Ag_3_PO_4_ has been studied and shown to have a high photo-oxidation capability. However, there is few reported example of a simple method to prepare Ag_3_PO_4_ coatings on various substrates. In this study a novel and simple method to immobilize Ag_3_PO_4_ on the surface of glass substrates has been developed. A silver phosphate paste based on a polyelectrolyte solution was applied to a smooth glass surface. The resulting dried material was calcined to obtain a coating that remained on the glass substrate. The coating layer was characterized by X-ray diffraction and energy dispersive X-ray spectrometry, and the optical band gap of the material was determined. The results indicated that an Ag_3_PO_4_ coating responsive to visible light was successfully prepared. The coating, under visible light irradiation, has the ability to decompose methylene blue. Although the coating contained some elemental silver, this did not adversely affect the optical band gap or the photocatalytic ability.

## Introduction

The development of sustainable energy utilization and environmental purification technologies is an important issue for ongoing development of society. In this context, research on photocatalysts is being actively conducted. Titanium dioxide TiO_2_, a typical inorganic solid photocatalyst, is widely used due to its excellent photocatalytic activity and ability to produce hydrogen by water splitting^1–5^. However, the development of photocatalysts that utilize visible light, which accounts for 43% of sunlight, has been studied as an effective means of solar energy use^3,4^. Photocatalysts driven by visible light are also important in the utilization of indoor light as an energy source^6^.

Recently, the photocatalytic activity of silver orthophosphate, Ag_3_PO_4_, has been investigated, and the compound has been shown to have a high photo-oxidation capability^7–12^. Photo-oxidative decomposition experiments using methylene blue revealed good photo-oxidative performance with a quantum yield of nearly 80%, which is tens of times more efficient than titanium dioxide or bismuth vanadate. In the photo-oxidative decomposition of water to generate oxygen, the performance of Ag_3_PO_4_ surpassed that of bismuth vanadate BiVO_4_ and tungsten oxide WO_3_ under visible light^7^. Although silver phosphate cannot be used for the conversion of water to hydrogen due to its slightly low conduction band potential, it has shown considerable promise for oxygen generation, decomposition of organic matter, and in antifouling applications^8^. In addition, recent studies have revealed the antimicrobial properties of materials containing silver phosphate^13^. For this reason, there have been numerous reports on the synthesis and utilization of Ag_3_PO_4_, especially since the report of Yi et al.^7^. Silver orthophosphate may be synthesized by precipitation^14–16^, ion exchange^15,17^, electrolysis^7^, and other methods^18,19^, each of which has its own advantages. Recently, silver phosphate crystals of various shapes^15,17–20^, core–shell particles^21,22^, and composites, all of which provide high photocatalytic activity, have been studied^10,12,23–30^.

The development of coating technologies for substrates is an important issue when using photocatalysts for surface antifouling applications^31^. Thin-film and thick-film technologies for semiconductors are also important in the development and advancement of electronic devices^32,33^. The immobilization of titanium dioxide, a typical photocatalyst, on the surface of materials has been the subject of much research and has been widely used in practical applications^5,34–37^.

There are various ways for producing semiconductor films, including the sol–gel method, vacuum deposition, sputtering, plasma CVD, and pulsed laser deposition. In the case of Ag_3_PO_4_, it is difficult to use an approach such as the sol–gel method to synthesize a coating film directly from a precursor solution on a solid surface, or a deposition method using plasma.

Noureen et al.^10^ investigated the antibacterial and photocatalytic properties of Ag_3_PO_4_/graphene oxide coated on cotton textiles. For these purposes, the coating has exhibited high performance. Furthermore, Xie et al.^38^ prepared titanium plates coated with polydopamine, graphene oxide, and Ag_3_PO_4_ and successfully eliminated biofilm on the metal surface. Nevertheless, these materials were coatings in which crystals were electrostatically deposited on a portion of the substrate surface. An example of fabrication for an Ag_3_PO_4_ coating that adheres to substrates is electrolytic deposition of silver plate, as reported by Yi et al.^7^. However, the substrate in this method is limited to a conductive medium such as silver plate.

The development of a simple method for the preparation of Ag_3_PO_4_ coatings on a variety of substrates may lead to more widespread use of Ag_3_PO_4_ as a highly efficient photocatalyst. In particular, if a method can be established for forming films on transparent materials such as glass, it will be possible to apply Ag_3_PO_4_ to optical devices and photoelectrodes, and this should contribute greatly to the development of optoelectronics using Ag_3_PO_4_. There are few reports for Ag_3_PO_4_ coating on transparent substrates. For example, Ma et al. successfully immobilized an Ag/Ag_2_O/Ag_3_PO_4_/Bi_2_WO_6_ photocatalyst on a glass surface^39^. Gunjakar et al.^40^ deposited Ag_3_PO_4_ on the surface of ITO substrate by chemical bath deposition method. For the utilization of Ag_3_PO_4_, it is desirable to discover more versatile coating methods.

In this study, we have developed a novel and simple method to immobilize Ag_3_PO_4_ as a coating on the surface of a glass substrate. The approach is based on a simple method for preparing titanium dioxide coatings for dye-sensitized solar cells via a precursor paste, but the composition of the paste is even simpler in the present application. This article is based on a study first reported in a short communication (in Japanese)^41^, to which substantial discussion and additions have been made.

In this paper, the coating layer was obtained by first preparing a paste, applying it to a glass surface, and then calcination. The obtained coating layer was subjected to scanning electron microscopy (SEM), energy-dispersive X-ray spectrometry (EDX), and X-ray diffraction (XRD) analyses. Also, the optical response of the samples was evaluated by measuring their diffuse reflection absorption spectra. Thermogravimetry (TG) and differential thermal analysis (DTA) were also performed to study the thermal reactivity of the dried paste.

## Materials and methods

### Materials

All the water used in the experiments was distilled once and then purified by ion exchange. Silver nitrate (Wako Pure Chemicals), disodium hydrogen phosphate (Kanto Chemical), carboxymethyl cellulose sodium salt (CMC-Na; Wako Pure Chemicals), and methylene blue (Wako Pure Chemicals) were used as received without purification. The commercial silver phosphate (Sigma-Aldrich), silver (Sigma-Aldrich), barium sulfate (Wako Pure Chemicals) were also used without further purification.

Silver orthophosphate was synthesized according to the method for producing silver phosphate fine particles reported by Khan et al.^16^. Specifically, 100 mL each of 0.020 mol L^−1^ aqueous silver nitrate solution and 0.020 mol L^−1^ aqueous disodium hydrogen phosphate solution were dropped simultaneously into 200 mL of stirred pure water. To remove the supernatant and obtain fine crystals, the resulting colloidal solution of Ag_3_PO_4_ was centrifuged. This procedure was repeated at least three times, until no precipitation of silver chloride occurred, whereupon sodium chloride solution was added to the supernatant. The resulting precipitate was dried under reduced pressure to obtain fine particles of Ag_3_PO_4_. The resulting powder was yellow in color.

**Figure 1:**
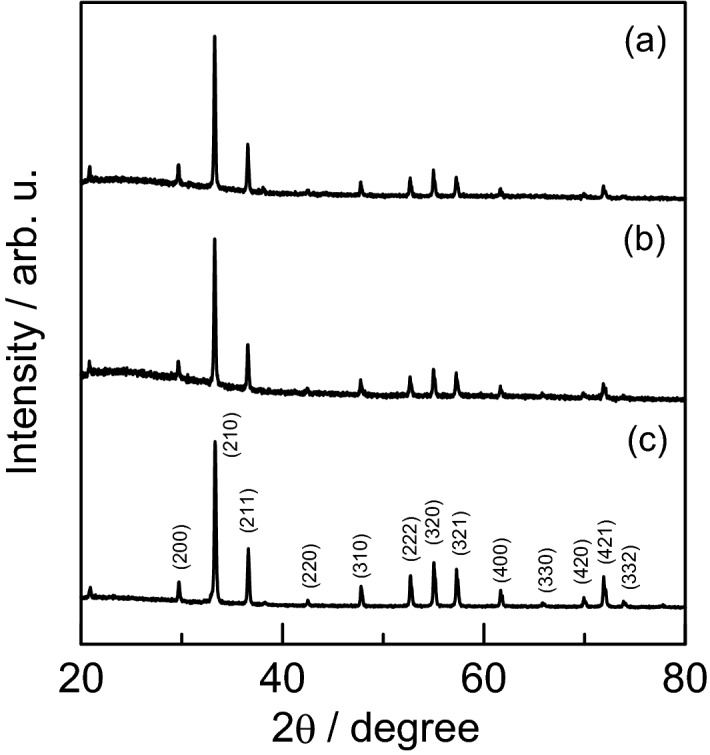
XRD patterns for samples synthesized at dropping rates of (**a**) 0.020 cm^3^ s^−1^ and (**b**) 0.33 cm^3^ s^−1^. For comparison, a diffraction pattern measured for (**c**) commercial silver phosphate is also shown.

To study the effect of the dropping rate on the product, the synthesis was attempted with two different dropping rates. The XRD patterns and SEM images for the obtained samples were measured. The XRD patterns for the samples synthesized at dropping rates of 0.020 cm^3^ s^−1^ and 0.33 cm^3^ s^−1^ are shown in Fig. 1. For comparison purposes, the diffraction pattern for commercial silver phosphate (Aldrich, > 99%) is also shown.

**Figure 2:**
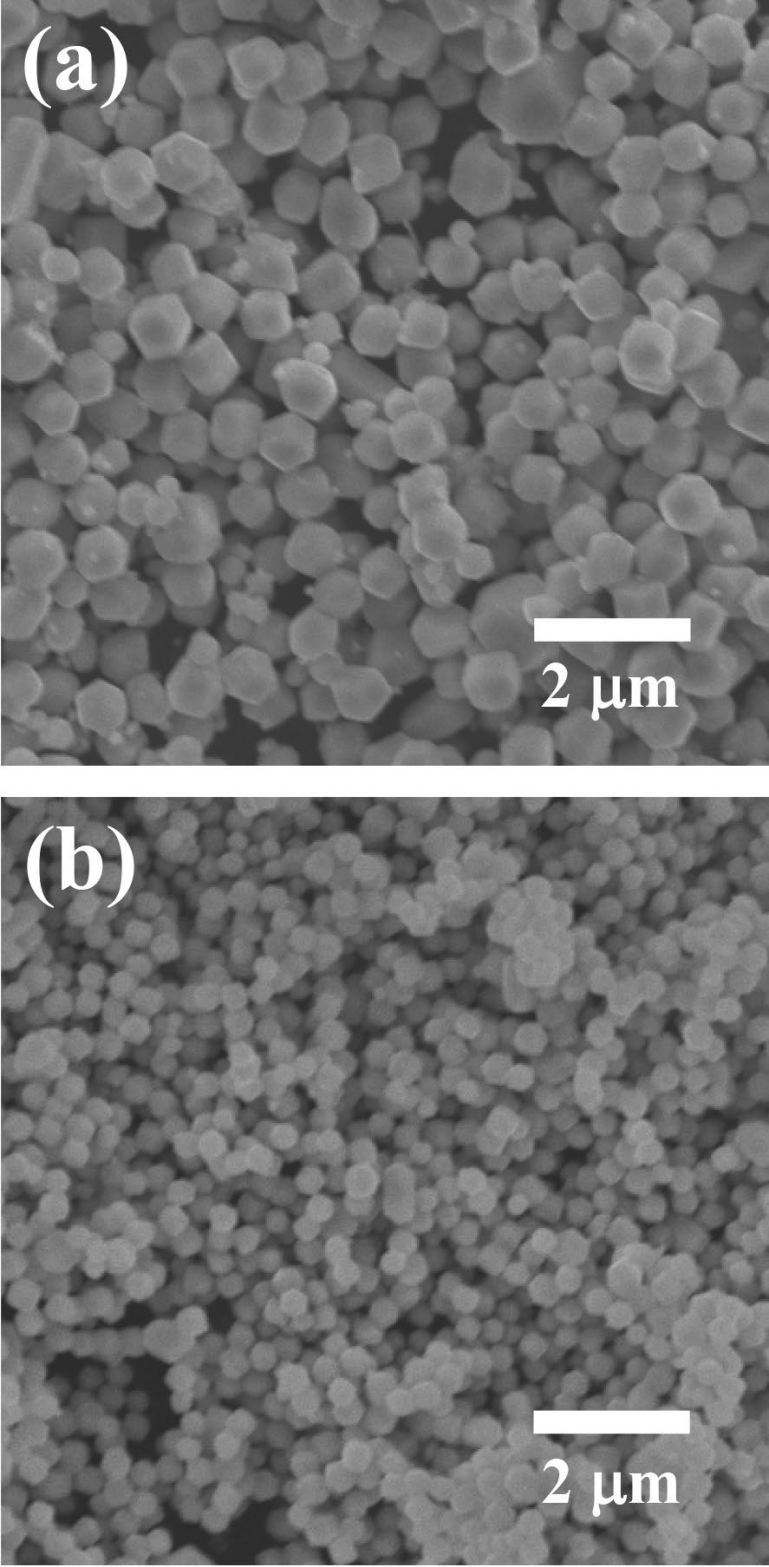
SEM images of microcrystals obtained for dropping rates of (**a**) 0.020 cm^3^ s^−1^ and (**b**) 0.33 cm^3^ s^−1^.

The samples obtained using the above method showed prominent diffraction peaks at 33.3° (210), 36.6° (211), 52.7° (222), 57.3° (321), and 55.0° (320)^22^. All of these major peaks were consistent with the commercial sample and assigned to the body-centered cubic structure of silver phosphate (JCPDS, card No. 6-505). The results indicated that the synthetic products were Ag_3_PO_4_. A slight overlap of the halo pattern indicated the presence of small amounts of amorphous or low-crystallinity product. The SEM images of the products obtained for each dropping rate are shown in Fig. 2. The crystal shape is hexagonal prismatic, which is consistent with the crystal structure of silver phosphate. The dropping rates of 0.33 cm^3^ s^−1^ and 0.020 cm^3^ s^−1^ yielded relatively uniform crystals of size ca. ~ 500 nm and ~ 1 µm, respectively. The particle size is smaller for synthesis at the increased dropping rate. The rapid dropping leads to rapid nucleation, resulting in an increase in crystal nuclei. This results in a smaller size of the yielded crystal. Subsequent studies were conducted using one of these microcrystals (dropping rate 0.33 cm^3^ s^−1^).

### Silver orthophosphate coatings on glass substrate

CMC-Na was used as a dispersion stabilizer and thickener in the preparation of silver phosphate paste. The Ag_3_PO_4_ obtained above, pure water, and CMC-Na were mixed in a mass ratio of 1:1:0.020–0.050, and a paste was prepared by kneading for 10 min while maintaining constant humidity. The CMC-Na and water were prepared in advance as an aqueous solution. A sufficient amount of the paste was then put onto a borosilicate glass substrate (18 × 18 mm) with both edges masked with 2.0 mm wide tape (3 M Scotch^TM^ Mending Tape, 58 µm thickness^42^). The paste was spread using a glass squeegee with a smooth surface to form a uniform paste layer on the substrate surface. The resulting paste layer was dried for 20 h at room temperature and under reduced pressure. The dried coatings were then sintered in an electric furnace at 300–500 °C for 2 h.

### Dye decomposition experiment for evaluation of photocatalytic activity

To examine the photocatalytic activity of the coatings, methylene blue degradation experiments were conducted under blue LED light irradiation. A glass substrate with the Ag_3_PO_4_ coating (as prepared in Sect. Silver orthophosphate coatings on glass substrate) was placed in 50 mL of a 20 mg L^−1^ methylene blue solution, placed in the dark, and stirred with a stirrer while aerating slowly. To observe only the decrease in concentration due to light irradiation of the sample, the adsorption of methylene blue on the sample was allowed to reach equilibrium. After reaching the adsorption equilibrium, the sample solution was irradiated with light from a blue LED light source. The absorbance of the sample solution at 665 nm was then measured at regular intervals. From the change in absorbance, the time course of the methylene blue concentration was measured. The blue LED used had a central emission wavelength of 462 nm and an irradiance density of 1.0 mW cm^−2^ (calculated as monochromatic light).

### Apparatus

Scanning electron microscopy was performed using a Hitachi S-3400 scanning electron microscope in backscattered electron mode under high vacuum. Elemental analysis in the scanning electron microscope was performed using an Ametek EDAX energy dispersive X-ray analyzer. Crystallographic analysis was performed using a Shimadzu XRD-6000 X-ray diffractometer with CuKα radiation (λ = 0.15418 nm). To investigate the optical response of the samples by diffuse reflectance spectral measurements, a JASCO V-560 UV–Vis-NIR absorption spectrophotometer equipped with a JASCO ISV-469 type integrating sphere was used. A solid sample cell with a quartz window was used for the measurements. Barium sulfate powder was used as the white light reference standard. A Shimadzu Biospec-1600 UV–visible absorption spectrophotometer was used for determination of the methylene blue concentration in aqueous solution. The concentration was determined using a calibration curve prepared from the absorbance of methylene blue standard solutions at 665 nm. For thermal analysis, a Shimadzu DTG-60H thermal analyzer was used to record the differential heat and thermogravimetric changes with increasing temperature.

## Results and discussion

### Formation of silver phosphate coatings on borosilicate glass substrate and characterization

First, the silver phosphate and CMC-Na solution were well mixed to prepare a paste. The paste was applied to a borosilicate glass plate and squeegeed to spread the paste uniformly. Silver phosphate paste with a mass ratio of silver phosphate, pure water, and CMC-Na of 1.0:1.0:0.010–0.050 was spread out and dried. For the range of 1.0:1.0:0.020–0.050, well-dried coatings adhered to the glass without cracks or voids were obtained. The coatings retained their structure on the glass surface even after sintering at 300 °C or 500 °C and washing the resulting coatings with distilled water. After sintering, there were no cracks in the coatings for specimens in the composition range 1.0:1.0:0.025–0.030, while cracks were observed for the specimens outside this range. It was also thought that it was necessary to study the process at a higher temperature. However, given that the melting points of orthophosphate Ag_3_PO_4_, pyrophosphate Ag_4_P_2_O_7_, and metaphosphate AgPO_3_ were reported to be 849 °C^43^, 585 °C^44^, and 482 °C^45^, respectively, and since a lower-temperature deposition process was also desirable from an energy standpoint, we proceeded with experiments in this temperature range. The thickness of the coating obtained by sintering the paste-casted substrate was determined by cross-sectional observation by SEM and the thickness was 20 ± 3 µm (n = 4). The thickness of the coating was smaller than the spacer thickness (3 M Scotch™ Mending Tape, 58 µm) used in paste application, because of shrinkage during drying and sintering.

**Figure 3:**
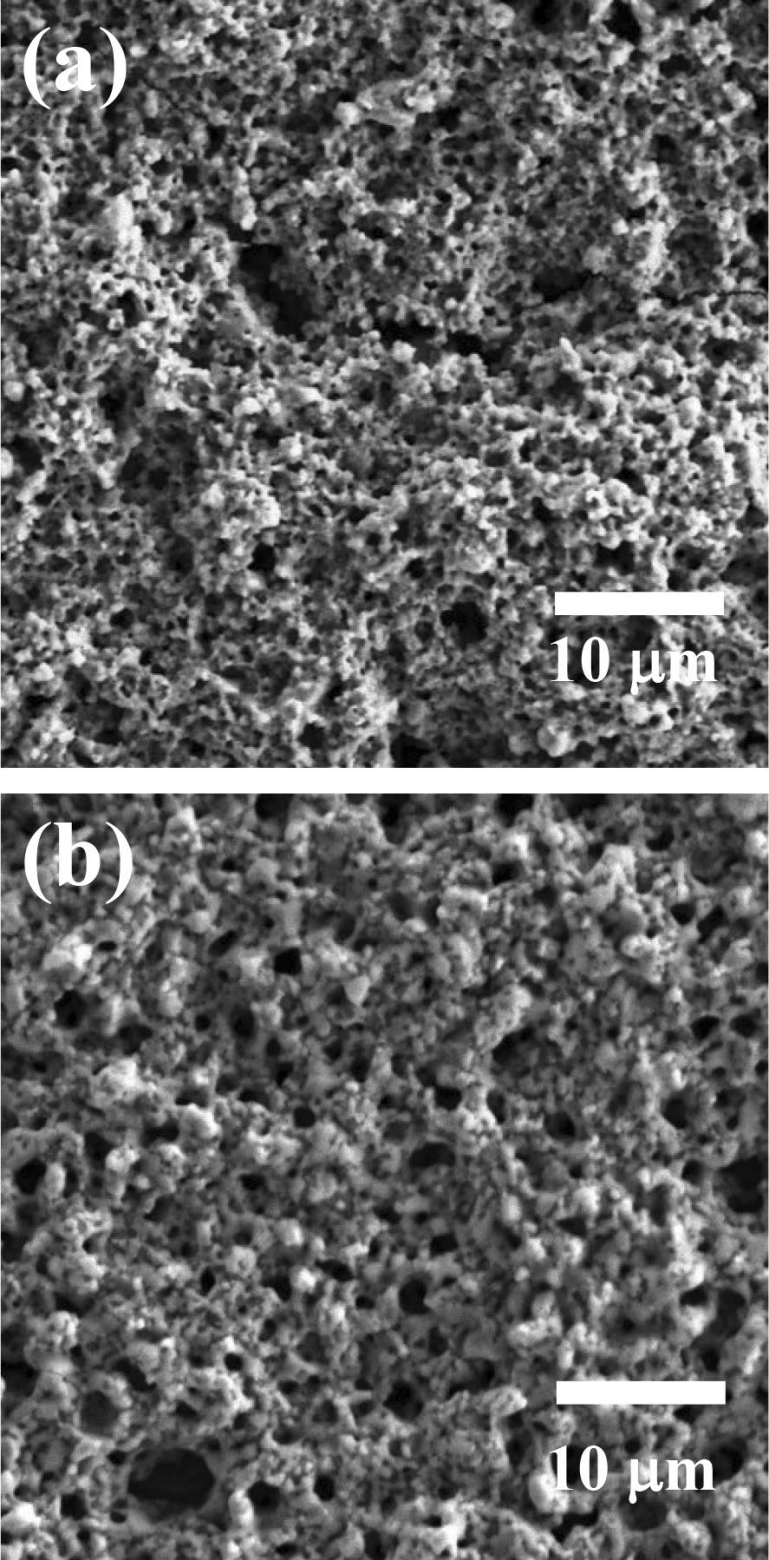
SEM images of surface of substrate coated with silver phosphate via calcination at (**a**) 300 °C and (**b**) 500 °C.

The results of characterization of the coating prepared using silver phosphate paste with a mass ratio of 1.0:1.0:0.030 (Ag_3_PO_4_:H_2_O:CMC-Na) are shown below. SEM images of the surface of the substrate coated with the paste and calcined at 300 °C (a) and at 500 °C (b) are shown in Fig. 3.

In the case of sintering at 300 °C (a), some particles were observed to remain, but the boundary between the particles was barely discernable due to coalescence. When the coating was sintered at 500 °C (b), the existence of particles did not be confirmed. However, a structure with a large number of pores in the micrometer to sub-micrometer size range was observed in both coatings after calcination. An EDX analysis of the coating during SEM revealed silver, phosphorus, oxygen, and a trace of sodium. The elemental abundance ratio for silver and phosphorus was approximately Ag/P = 3, which is consistent with the stoichiometry of silver phosphate.

**Figure 4:**
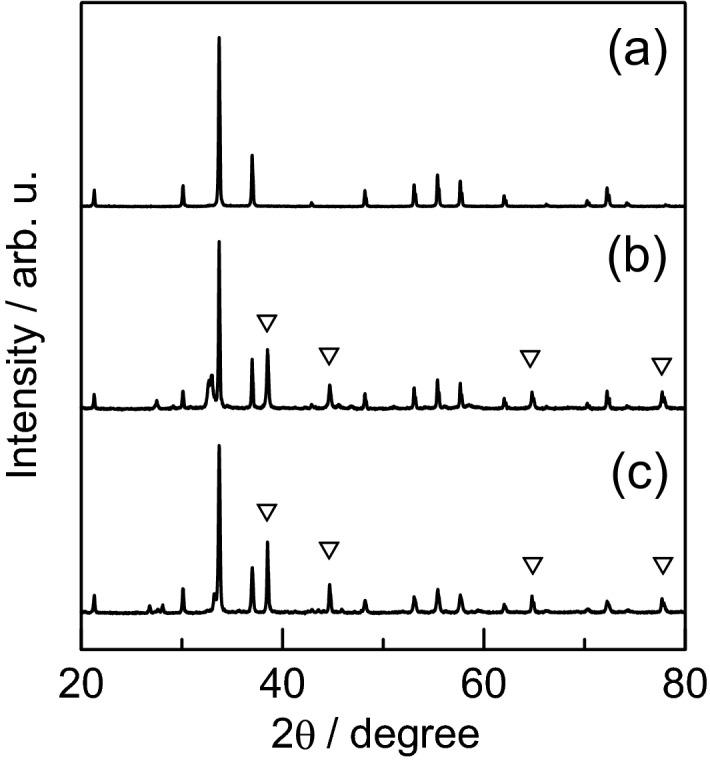
XRD patterns for silver phosphate coating obtained by calcination at (**b**) 300 °C and (**c**) 500 °C. An XRD pattern for (**a**) the dried paste before calcination is also shown.

The XRD patterns for the silver phosphate coating obtained by calcination are summarized in Fig. 4, the patterns for calcination at 300 °C and 500 °C being shown in Fig. 4b, c, respectively. For comparison purposes, the XRD pattern for the dried paste before calcination is also presented (Fig. 4a). The diffraction intensities are normalized with respect to the intensity of the largest diffraction peak.

In the XRD pattern for the dried paste before calcination, only the peak for silver phosphate was identified. However, peaks due to elemental silver (JCPDS, card no. 04-0783), indicated by the inverted triangle marks, were also observed for the samples calcined at 300 °C and 500 °C. This is attributed to the thermal reduction of silver phosphate or some silver compound generated during calcination. The only silver source is silver phosphate, however since CMC-Na is added, complexes of silver and CMC may also be involved. In the sample calcined at 300 °C, there is a peak at around 32° that is not seen for the other samples. The diffraction peaks were compared with the XRD data of CMC-Na, several polymorphs of carbon, and several silver salts. And the most consistent with silver (I) oxide^46^. Since silver oxide decomposes into oxygen and silver at about 300 °C, the fact that this peak disappeared upon calcination at 500 °C also supports this identification^47^.

**Figure 5:**
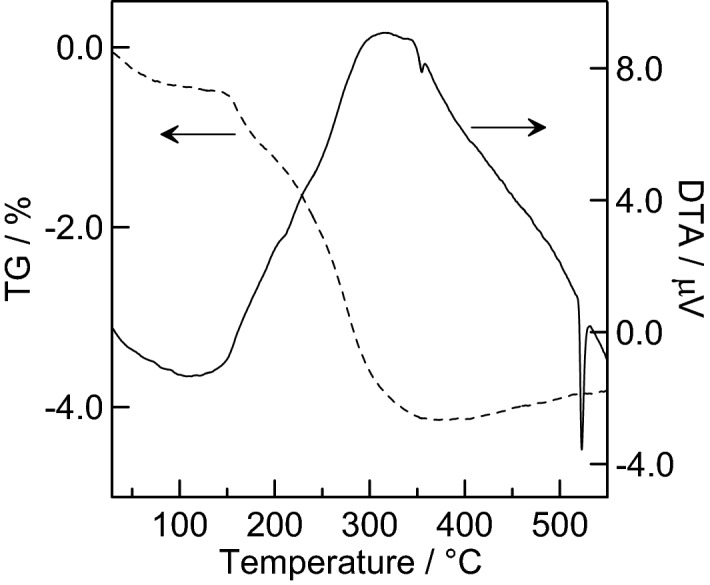
Results of differential thermal analysis (solid line) and thermogravimetry (dashed line) of a silver phosphate paste.

Thermogravimetry (TG, dashed line) and differential thermal analysis (DTA, solid line) curves measured for dried silver phosphate paste are presented in Fig. 5. The measurements were performed on a paste that had been dried on a glass substrate and was then scraped off with a metal scraper.

The curves indicate that a decrease in weight up to a heating temperature of around 350 °C occurred, and the weight increased slightly above 350 °C. This mass loss was attributed to the removal of water and the CMC-Na contained in the dry paste as a result of the heating. Dhanabal et al.^48^ reported the results of a TG–DTA analysis of silver phosphate synthesized by precipitation and hydrothermal treatment. They reported that a small endothermic peak corresponding to the partial melting of Ag_3_PO_4_ was observed at 531 °C or 526 °C depending on the synthesis conditions. In the TG–DTA analysis in the present study, an endothermic peak was also observed at 523 °C. In addition to this, an endothermic peak at 355 °C was noted. This latter peak may also correspond to partial melting of the phosphate. Another endothermic peak was detected at a lower temperature, even though the particle size of the phosphate was almost the same as that synthesized by Dhanabal et al.^48^. This is due to coordination of the carboxyl groups of CMC-Na with silver. However, the influence of the coexistence of sodium ions cannot be ruled out.

**Figure 6:**
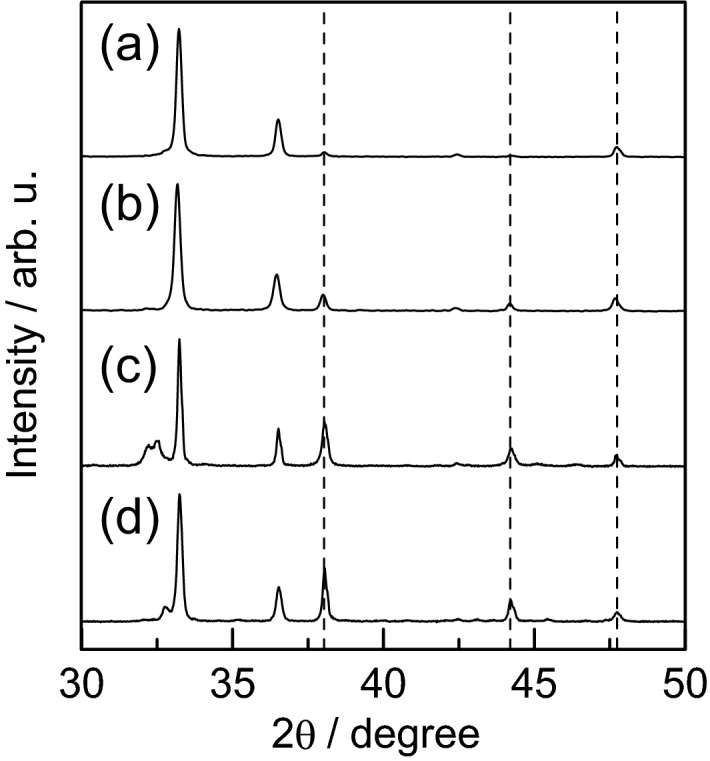
XRD patterns for raw silver phosphate calcined at (**a**) 300 °C, (**b**) 500 °C and silver phosphate paste calcined at (**c**) 300 °C, (**d**) 500 °C. Here, the composition ratio for silver phosphate paste is 1.0:1.0:0.030 (Ag_3_PO_4_:H_2_O:CMC-Na). The dashed vertical line represents the diffraction angle for elemental silver.

Next, an experiment was conducted to investigate the cause of silver formation during heating of the paste. The XRD patterns were measured for samples of silver phosphate paste with a mass ratio of 1.0:1.0:0.030, which were calcined at 200–500 °C, and ground in an agate mortar. The XRD patterns for raw silver phosphate calcined at 200–500 °C were also measured and compared. The results are shown in Fig. 6.

The strongest peak for elemental silver appears near 38°. The peak did not appear in the diffraction pattern if silver phosphate was only calcined at 300 °C, and it was only slightly observed at 500 °C. In contrast, in the presence of CMC-Na, the peak of 38° was observed in both 300 °C and 500 °C.

**Figure 7:**
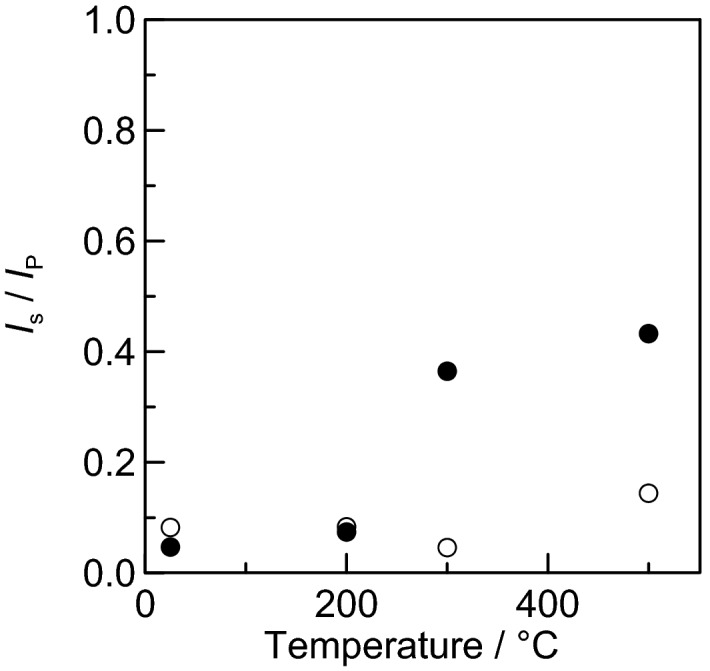
Plot showing the correlation between *I*_S_/*I*_P_ and the calcination temperature. Here, the intensity *I*_S_ of the diffraction peak near 38° is for metallic silver, and the intensity *I*_P_ of the diffraction peak near 33° is for silver orthophosphate. Open circles indicate data for raw silver phosphate, and closed circles indicate data for the paste (mixture with CMC-Na).

The intensity of the diffraction peak around 38° due to elemental silver is defined as *I*_S_, and the intensity of the diffraction peak around 33° due to silver phosphate is defined as *I*_P_. The ratio *I*_S_/*I*_P_ was determined and plotted against temperature as shown in Fig. 7. The increase in the *I*_S_/*I*_P_ ratio at high temperatures is significantly larger with the presence of CMC-Na. This result clearly shows that CMC-Na plays a positive role for silver formation, although silver formation by direct heating cannot be ruled out.

**Figure 8:**
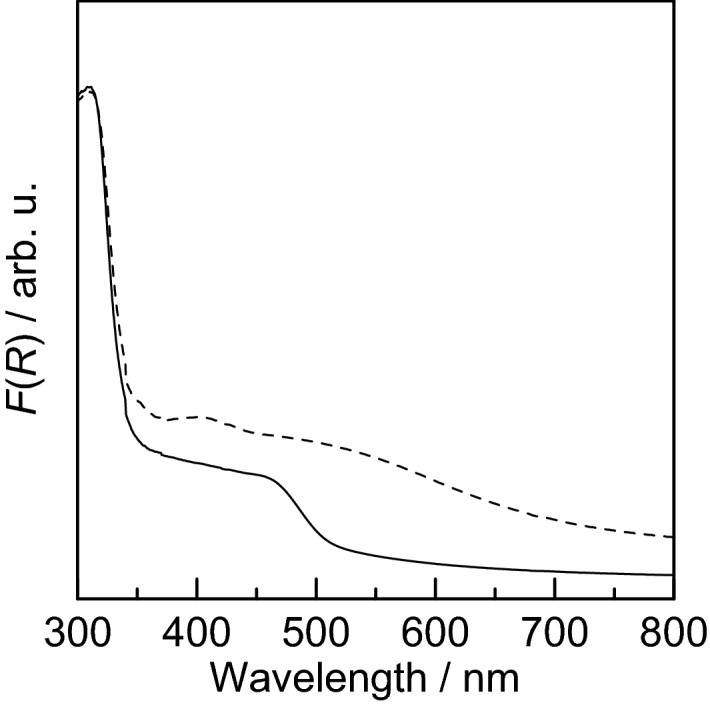
Wavelength dependence of *F*(*R*) for coatings obtained by calcination at 300 °C (dashed line) and 500 °C (solid line).

The diffuse reflectance absorption spectra of the samples were measured to characterize the photoresponsive properties of the coatings and to determine the optical band gap. The specific reflectance *R* = *R*_S_/*R*_Ref_ was obtained from the reflectance *R*_S_ for each sample and the reflectance *R*_Ref_ for the standard material, barium sulfate. The KM function *F*(*R*) was calculated from the *R* value using the Kubelka–Munk Eq. (1):^49^

1 $$F\left( R \right){\text{ }} = K/S = {\text{ }}\left( {{\text{1}} - R} \right)^{{\text{2}}} /{\text{2}}R$$

where *K* is the absorption coefficient and *S* is the scattering coefficient. The wavelength dependence of *F*(*R*) is shown in Fig. 8 for the coatings obtained by calcination at 300 °C (dashed line) and 500 °C (solid line).

Both coatings showed a wide range of absorption over the UV to visible region. The absorption in the visible region extended to nearly 500 nm. This finding is consistent with the appearance of the samples. Therefore, the coatings obtained in this study are confirmed to be responsive to visible light and are expected to exhibit photocatalytic activity in this wavelength range.

**Figure 9:**
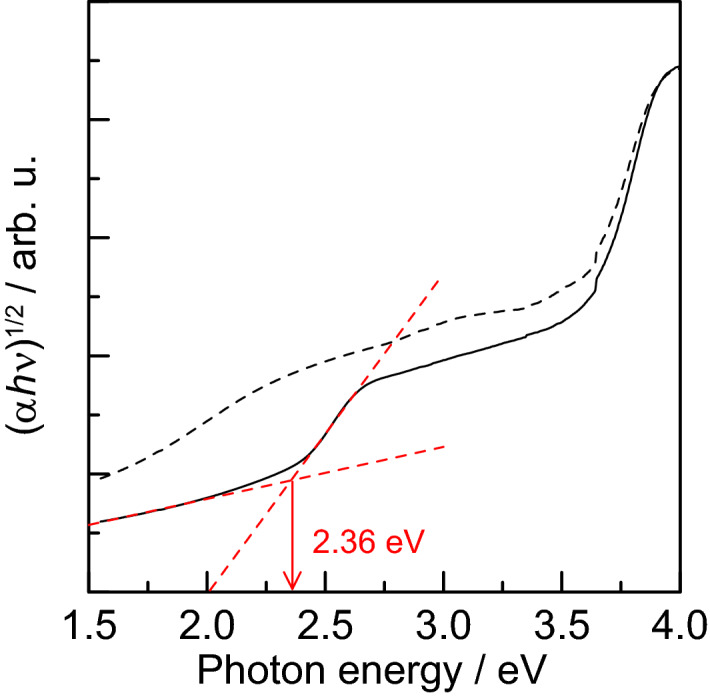
Dependence of (*αhν*)^1/2^ on photon energy *hν* (Tauc plot) for samples sintered at 300 °C (dashed line) and 500 °C (solid line).

The band gaps of the coatings obtained by calcination at 300 °C and 500 °C were estimated using a Tauc plot^50^, which is a plot of (*αhν*)^*n*^ against the photon energy *hν*, where *α* is the absorption coefficient of the material. For band-gap transitions of silver phosphate, *n* = 1/2 because they are indirectly allowed transitions^51,52^. Although it is difficult to measure the absolute value of the absorption coefficient *K* by conventional spectral reflectance measurements, it is assumed that the scattering coefficient *S* is constant and *α* = *F*(*R*). The Tauc plot obtained in this study is shown in Fig. 9.

For the coating calcined at 500 °C, a clear edge appeared around 2.5 eV. From the intersection of tangental lines (red dashed line) drawn on the absorption edge and the baseline, the band gap energy was estimated to be 2.36 eV. Yi et al.^7^ reported the band gap of Ag_3_PO_4_ to be 2.4–2.5 eV. Opoku et al.^53^ estimated the band structure of bulk Ag_3_PO_4_ by first-principles calculations and reported a value of 2.43 eV as the indirect band gap. The estimated band gap of 2.36 eV results in good agreement with these values. However, the coating calcined at 300 °C did not show a clear edge in the visible region. This is due to the formation of carbon and silver compounds derived from CMC-Na during calcination at low temperatures, which raise the baseline. Therefore, the band gap of this sample did not estimated because an absorption edge of Ag_3_PO_4_ did not be identified.

The results of the present experiments confirm the photocatalytic activity of silver phosphate as outlined below. The results clearly demonstrate that the present coating exhibits light absorption in the visible region and promising photocatalytic activity.

Clearly, a silver phosphate coating can be prepared from a simple paste consisting of silver phosphate and CMC-Na from these results, The coating has a large number of micropores on its surface and is responsive in the visible light region. Although the precursor paste used in this study contained only a polyelectrolyte as a dispersant and no inorganic ultrafine particles as a sintering additive, an excellent coating was successfully prepared. This is due to the low melting point of Ag_3_PO_4_, which readily self-sintered and recrystallized upon heating, resulting in the disappearance of the particle interfaces. The coexistence of CMC-Na had the effect of enhancing the ease of this change. We found that the coating contained a small amount of metallic silver due to the coexistence of CMC-Na. For a semiconductor deposition process, it is desirable to have a single-phase composition. However, a single phase is not always the best from the perspective of photocatalytic activity. For example, Zhu et al.^21^ prepared an Ag_3_PO_4_ photocatalyst contained metallic silver, and reported that its photocatalytic ability was higher than that of Ag_3_PO_4_ alone. Zhang et al.^20^ also reported that the coexistence of graphite carbon nitride enhances the photocatalytic performance of Ag_3_PO_4_. There have been many reports on the enhancement of photocatalytic activity by plasmonic effects in combination with silver. Dong et al.^54^ succeeded in improving the performance of an Ag_3_PO_4_ photocatalyst by a simple sintering method. The elemental silver produced by calcination is beneficial for the rapid transfer of photoexcited electrons of Ag_3_PO_4_ and inhibits the photocorrosion of Ag_3_PO_4_, thereby improving the stability of the photocatalyst. It is also assumed that elemental silver is produced during sintering because Ag^+^ in the Ag_3_PO_4_ semiconductor is reduced by thermally excited electrons. The aforementioned researchers reported the formation of elemental silver by calcination of pure silver phosphate. This may seem to contradict the results shown in Fig. 6. However, even in the report by Dong et al., no peaks for elemental silver appeared in the XRD pattern due to calcination of silver phosphate, and our results are consistent with this. The coexistence of CMC-Na salt facilitated the formation of silver, which is attributed to the effect of carboxy groups coordinating with ionic silver to lower its melting point. The phenomenon that CMC lowers the melting point of silver-related substances and facilitates the formation of silver has been reported by Miyama et al.^55^.

### Dye decomposition experiment for evaluation of photocatalytic activity

**Figure 10:**
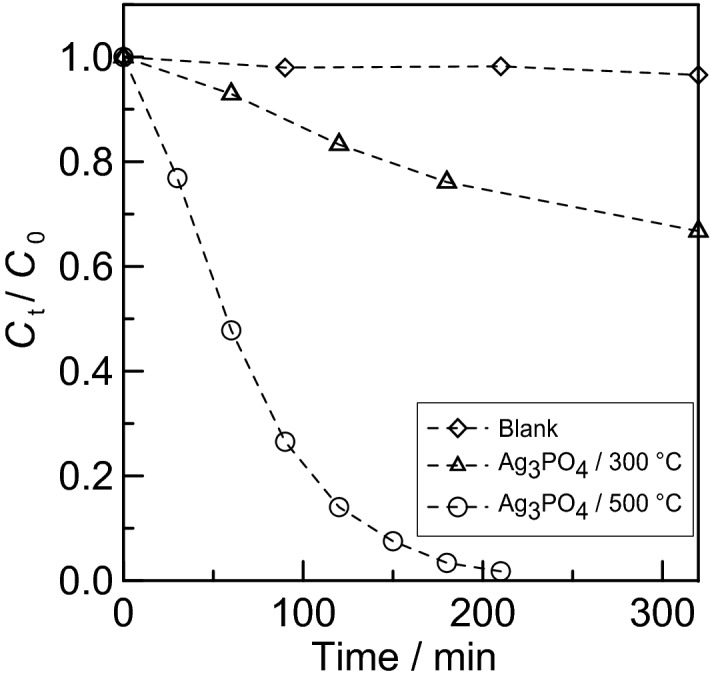
Time course of specific concentration *C*_t_/*C*_0_ of methylene blue under blue LED illumination for (1) substrate only, (2) silver phosphate coated substrate (calcined at 300 °C), and (3) silver phosphate coated substrate (calcined at 500 °C). Here, *C*_0_ is the initial concentration of methylene blue and *C*_t_ is the concentration at time *t*.

The decomposition of organic dyes was attempted by irradiating the coated sample with blue LED light in a methylene blue solution. By examining the degradability of organic dyes, the photocatalytic activity during visible light illumination can be confirmed. One glass substrate with a coating was placed in 50 mL of 20 mg L^−1^ methylene blue aqueous solution and allowed to reach adsorption equilibrium in the dark. After that, the methylene blue was irradiated with a blue LED (central wavelength 462 nm, 1.0 mW cm^−2^) and the absorbance of the aqueous solution was measured at regular intervals to confirm whether the methylene blue had decomposed. The results are shown in Fig. 10. Here, *C*_0_ is the initial concentration and *C*_t_ is the concentration at time *t*.

Obviously, only the Ag_3_PO_4_ coating caused a significant decrease in methylene blue concentration under visible light irradiation. This suggests that the methylene blue was decolorized or decomposed by the photocatalytic reaction using the coating under visible light. This clearly indicates that the coating obtained in this study possesses photocatalytic activity under visible light irradiation. Fitting the data to a single exponential decay curve, the rate constant and time constant for decomposition by the coating sintered at 500 °C were estimated to be 0.0146 min^−1^ (2.45 × 10^–4^ s^−1^) and 68.2 min (4.09 × 10^3^ s), respectively. In contrast, the coating at 300 °C exhibited slower decomposition than that at 500 °C. The coating at 300 °C contains impurities and defects, as discussed above. The impurities and defects promoted carrier recombination, resulting in low photocatalytic efficiency^56^.

Gunjakar et al.^40^ prepared Ag_3_PO_4_-deposited ITO substrates by chemical bath deposition route and studied the degradation of methylene blue by the substrates. A 2 cm^2^ substrate with 0.9 mg cm^−2^ of Ag_3_PO_4_ deposited per area was immersed in 3 mL of methylene blue solution, and irradiated with visible light (> 420 nm). As a result, the methylene blue decomposed in approximately 1 h. Although the initial concentration of methylene blue did not found in this literature, the first order decomposition rate constant is estimated to be 0.017–0.050 min^−1^ (2.8–8.3 × 10^–4^ s^−1^). The result was comparable to the findings of present study.

The comparison with a previous report above shows that the coating has sufficient degradation efficiency, nevertheless the effect of silver coexistence in the coating is not clear. Therefore, a comparison with previous reports on the composite Ag/Ag_3_PO_4_ was conducted. Piccirillo et al.^57^ studied the degradation of methylene blue by Ag/Ag_3_PO_4_ sample prepared by ion exchange of calcium phosphate with silver ion. As a result, the first-order decomposition rate constant was determined to be 9.8–12.8 × 10^2^ h^−1^ (0.27–0.36 s^−1^). In this experiment, the initial concentration of methylene blue was 5 mg L^−1^, the concentration of photocatalyst in the slurry was 0.25 g L^−1^, and a visible lamp of 50 W m^−2^ (5 mW cm^−2^) was used as the light source. Liu et al.^58^ synthesized Ag/Ag_3_PO_4_ by solid phase reaction and subsequent photoirradiation, and also attempted to degrade methylene blue. The first-order decomposition rate constant was determined to be 0.376–0.713 min^−1^ (0.0063–0.012 s^−1^). In the report, the initial concentration of methylene blue was 20 mg L^−1^, the concentration of photocatalyst in the slurry was 3 g L^−1^, and the light source was a 450 W Xe lamp with a cutoff for light below 420 nm. The rate constants obtained in our experiments are about one order of magnitude smaller than those of Liu et al. Although the initial concentration of methylene blue in our experiments was about the same as 20 mg L^−1^, the amount of photocatalyst on the glass substrate was small (ca. 4.5 mg cm^−2^, 2.9 cm^2^), and we used a weak LED light source with long wavelength (462 nm, 1.0 mW cm^−2^). These results indicate that the decomposition reaction is not extremely slow, but rather comparable. From this, we conclude that the coexistence of silver did not adversely affect the decomposition ability of methylene blue. Meanwhile, we found also no evidence that the coexistence of silver accelerated the degradation. It is necessary to conduct experiments under the same conditions using samples with controlled silver production to reveal the effect of silver coexistence. Since much study is needed to control the amount of silver produced, further research is now in progress.

**Figure 11:**
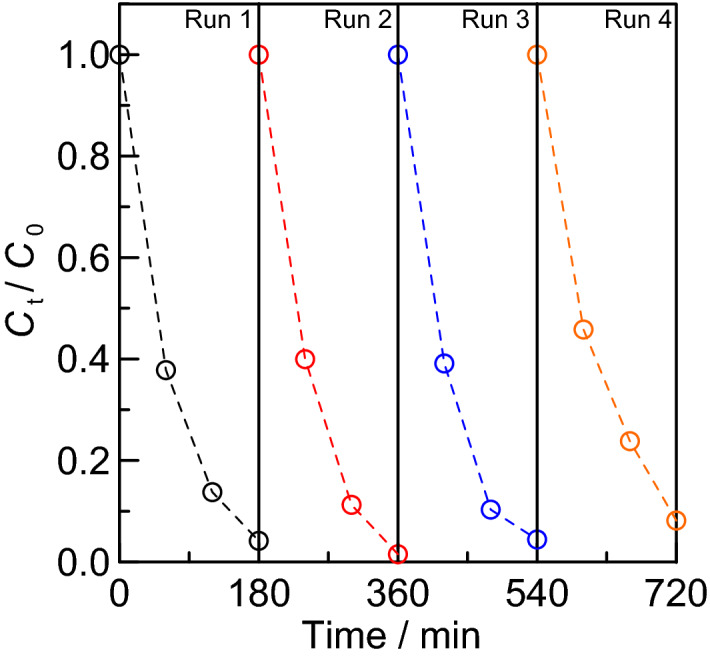
Time course of specific concentration *C*_t_/*C*_0_ of methylene blue under blue LED illumination for silver phosphate coated substrate (calcined at 500 °C) during the recycling test. Here, *C*_0_ is the initial concentration of methylene blue and *C*_t_ is the concentration at time *t*.

The reusability of the catalyst is one of the most important parameters of its application as heterogeneous photocatalyst. For this reason, the reusability of the prepared Ag_3_PO_4_ coating as a photocatalyst was examined. After decomposing methylene blue under visible light irradiation as in Fig. 10, the coating was placed in a new methylene blue solution and exposed to light again. The time course of the methylene blue concentration is shown in Fig. 11.

In four repeated trials, most of the methylene blue was photocatalytically degraded. This indicates that the coating has a high photocatalytic capacity and maintains it during repeated trials. Katsumata et al.^59^ evaluated the stability of Ag_3_PO_4_ photocatalyst by repeated experiments of bisphenol-A degradation. The results showed that the photocatalytic activity of Ag_3_PO_4_ was effectively maintained even after five recycling runs. Our results are in agreement with this report. The results of this experiment indicate that the prepared Ag_3_PO_4_ coating can be reused as a photocatalyst.

## Conclusion

In this study, a novel and simple method for immobilizing Ag_3_PO_4_ on the surface of glass substrates was developed. A paste consisting of Ag_3_PO_4_, water, and polyelectrolyte was applied to the glass surface, dried, and then calcined to obtain a coating that remained on the glass substrate. The coating layer was characterized by XRD and EDX, and the main crystal phase of the coating was confirmed to be Ag_3_PO_4_. Then, the visible light responsivity of the coating was evaluated by diffuse reflectance spectral measurement and decomposition of organic dyes under visible light irradiation. The results showed that the coating was responsive to visible light and showed degradation activity for organic dyes. The coating contains elemental silver generated during the sintering process, and this origin was also examined. For this purpose, the effects of the presence of CMC-Na and calcination temperature on silver formation were studied, and the results clearly show that CMC-Na plays an active role in the formation of silver, although silver formation by direct heating also occurs.
